# Supplementary figures and images for: Upper extremity joint tenderness as a practical indicator for assessing presenteeism in rheumatoid arthritis patients: A cross-sectional observational study
Source: PLoS One. 2025 Jun 5;20(6):e0318047. doi: 10.1371/journal.pone.0318047 (PMC12140210; doi:10.1371/journal.pone.0318047)

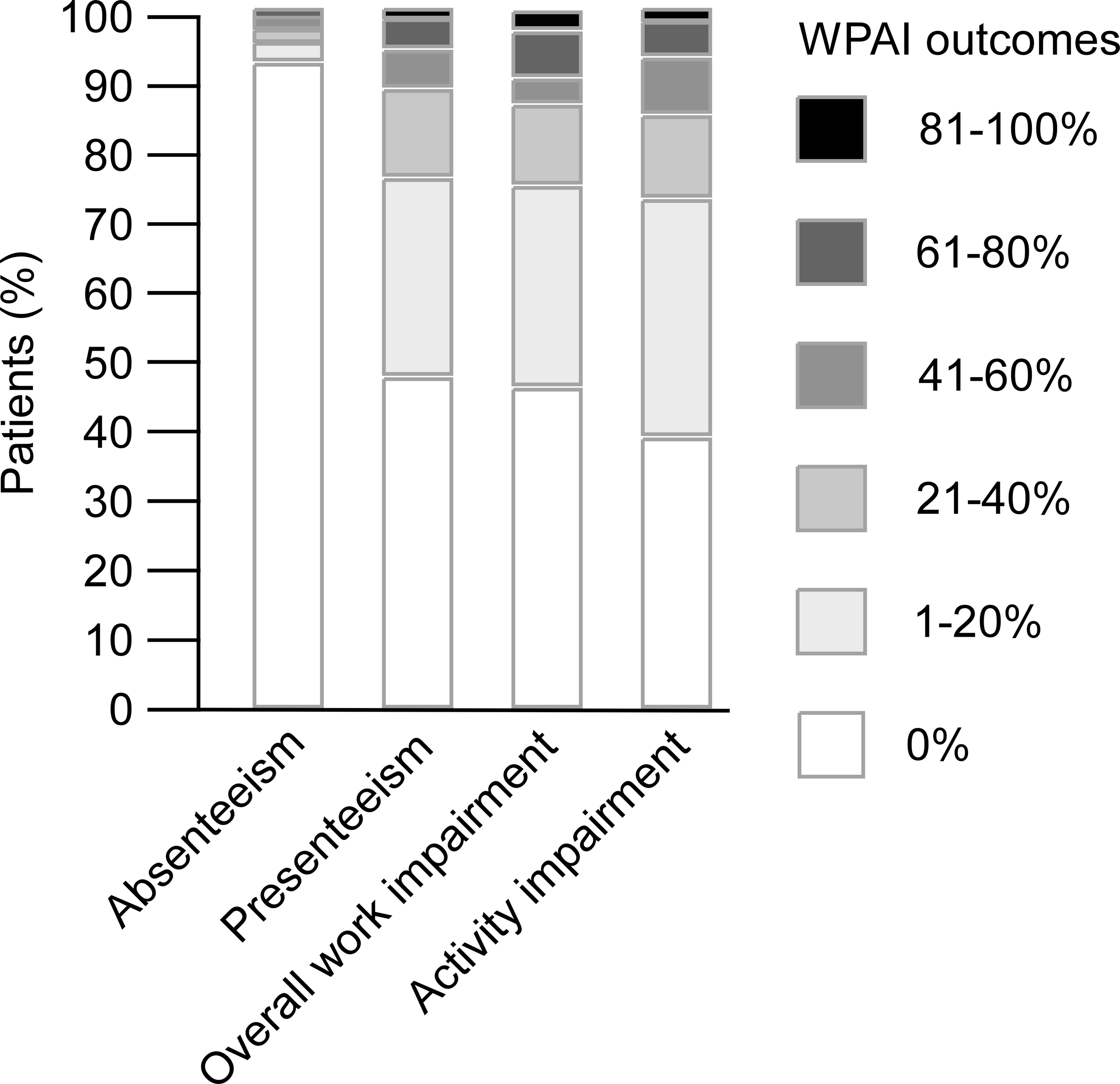

Supplement: S1 Fig — The vertical axis shows the percentages of patient, with each WPAI outcome displayed separately and shaded in grayscale. (TIF) [file pone.0318047.s001.tif]
