# Supplementary material for: Upper extremity joint tenderness as a practical indicator for assessing presenteeism in rheumatoid arthritis patients: A cross-sectional observational study
Source: PLoS One. 2025 Jun 5;20(6):e0318047. doi: 10.1371/journal.pone.0318047 (PMC12140210; doi:10.1371/journal.pone.0318047)
Supplement: S1 Table — (DOCX) [file pone.0318047.s002.docx]

| **S1 Table. The association of upper and lower extremity TJC as binary variables with the percentage of presenteeism.** | | | | |
| --- | --- | --- | --- | --- |
| Independent variables | Univariate | | Multivariate (n=199) | |
|  | β value estimate (95% CI) | *P* | β value estimate (95% CI) | *P* |
| Upper extremity TJC (>0 vs 0) | 16.7 (10.8, 22.6) | <0.001 | 9.33 (3.48, 15.2) | 0.002 |
| Lower extremity TJC (>0 vs 0) | 13.3 (6.21, 20.4) | <0.001 | 1.78 (-4.74, 8.29) | 0.591 |
| Upper extremity SJC (>0 vs 0) | 11.2 (4.69, 17.6) | 0.001 |  |  |
| Lower extremity SJC (>0 vs 0) | 19.2 (9.72, 28.6) | <0.001 |  |  |
| Multivariate model: *R*^2^ = 0.41. CI: confidence interval. See Table 1 for other abbreviation definitions. The values in the multivariate analysis are adjusted for the confounding effects of the following variables: age (years), gender, disease duration (years), HAQ-DI, ESR (mm/h), oral steroid use, and bDMARDs or tsDMARDs. | | | | |
