# Supplementary material for: Upper extremity joint tenderness as a practical indicator for assessing presenteeism in rheumatoid arthritis patients: A cross-sectional observational study
Source: PLoS One. 2025 Jun 5;20(6):e0318047. doi: 10.1371/journal.pone.0318047 (PMC12140210; doi:10.1371/journal.pone.0318047)
Supplement: Attachment — Submitted filename: Response to Reviewers R1.docx [file pone.0318047.s005.docx]

**Response to reviewers’ comments**

**Reviewer #1:**

We sincerely thank Reviewer #1 for the positive feedback and for recognizing the clinical implications of our study. We appreciate the constructive suggestions and have addressed them accordingly.

**Comment 1**

**The abstract does not reflect the study completely. Please include the following lines in abstract**

**1. Please mention in methods that presenteeism was ‘self-reported’**

**2. Please mention in the results that a positive correlation was seen between upper extremity TJC and presenteeism but not with lower extremity TJC**

**3. Please change the last line in conclusion of abstract to ‘Minimizing TJC in the**

**upper extremities, particularly in the shoulders and fingers, could be important**

**treatment goal to reduce work disability in RA patients.’**

**Response:** As suggested by Reviewer #1, we have revised the abstract as follows:

1. We have clarified that presenteeism was ‘self-reported’ in the “Methods” section of the “Abstract” (Page 2, Line 11).

2. We have revised the “Results” section of the “Abstract” to indicate that a positive correlation was seen between upper extremity TJC and presenteeism, but not with lower extremity TJC (Page 3, Lines 2–3).

3. We have modified the last sentence of the “Conclusion” of the “Abstract” to: ‘Minimizing TJC in the upper extremities, particularly in the shoulders and fingers, could be an important treatment goal to reduce work disability in RA patients.’ (Page 3, Lines 5 and 6).

**Comment 2**

**Please summarise the study in conclusion in the manuscript in a better way.**

**Response:** As suggested by Reviewer #1, we have revised the conclusion to provide a clearer and more concise summary of our findings. Specifically, we have emphasized the significance of upper extremity TJC, particularly in the shoulders and fingers, as a predictor of work disability in RA patients.

**Comment 3**

**Please mention the average interval between collecting WPAI data for 158 patients that were included in the temporal change comparison.**

**Response:** Reviewer #1 mentioned 158 patients; however, the temporal change analysis included 137 patients who continued working, after excluding 21 retirees. Therefore, we have reported the average interval for the 137 patients as 248 ± 121 days in the manuscript and have also made slight modifications to the surrounding text for clarity and coherence (Page 16, Lines 10 and 11).

**Reviewer #2:**

We sincerely thank Reviewer #2 for the positive feedback and for recognizing the value of our study. We greatly appreciate Reviewer #2’s kind words regarding the title, abstract, and introduction.

**Specific point**

**However, the cross-sectional design limits the ability to establish causality. To strengthen the analysis, the authors may consider incorporating a multivariable model that adjusts for socioeconomic status, as this could be an important confounder in assessing work disability. Overall, this is an impressive manuscript that contributes to the existing literature on the subject.**

**Response:** We sincerely appreciate Reviewer #2’s valuable comments, particularly regarding the potential confounding effect of socioeconomic status (SES) on work disability. As we mentioned in the "Discussion" section (the first limitation comment on Page 20, Lines 13-17), we acknowledge this limitation and the need for cautious interpretation of our results. Unfortunately, our cohort data does not include indicators of SES which have been reported as relevant factors in the association with work disability. Additionally, due to the lack of ethical approval to collect SES data from the facility, we were unable to incorporate this information into our analysis. However, although adjustments for SES as a confounder are not included, we believe that our data still offer meaningful insights into the relationship between upper extremity TJC and work disability in RA patients, contributing to a broader understanding of this topic.

Furthermore, as suggested by Reviewer #2 regarding the limitations of the cross-sectional study design, we have revised the manuscript to clearly acknowledge this issue. Specifically, we have noted that the cross-sectional nature of our study limits the ability to establish causality and requires careful interpretation (Page 22, Lines 9-11).

We sincerely appreciate your insightful suggestions, and we hope that our responses adequately address your concerns.
